# The effectiveness of Nurture and Play: a mentalisation-based parenting group intervention for prenatally depressed mothers

**DOI:** 10.1017/S1463423619000914

**Published:** 2019-12-16

**Authors:** S.J. Salo, M. Flykt, J. Mäkelä, Z. Biringen, M. Kalland, M. Pajulo, R.L. Punamäki

**Affiliations:** 1 Helsinki University, Helsinki, Finland; 2 Tampere University, Helsinki, Finland; 3 National Institute for Health and Welfare, Helsinki, Finland; 4 Colorado State University, US; 5 Turku University, Helsinki, Finland

**Keywords:** depressive symptoms, early intervention, emotional availability, reflective functioning

## Abstract

**Aim:**

This randomised control trial (RCT) study examined the effectiveness of a mentalisation-based perinatal group intervention, Nurture and Play (NaP), in improving mother–infant interaction quality and maternal reflective functioning and in decreasing depressive symptoms.

**Background:**

Few preventive prenatal interventions have been developed for primary health care settings for mothers with depressive symptoms. Furthermore, previous prenatal intervention studies have only concentrated on reducing depressive symptoms and have not directly addressed enhancing optimal parenting qualities.

**Methods:**

The participants were 45 pregnant women with depressive symptoms. Women in the randomly assigned intervention group (*n* = 24) participated in the manualised, short-term NaP intervention group from pregnancy until the baby’s age of seven months, whereas control group women received treatment as usual (TAU). Maternal emotional availability (EA), reflective functioning (RF) and depressive symptoms were measured before the intervention and at the infants’ 12 months of age, and changes were evaluated using repeated measure analyses of variances (ANOVAs).

**Findings:**

The results showed that the intervention group displayed higher maternal sensitivity and RF and more reduction in depressive symptoms than the control group when babies were 12 months old. These findings provide preliminary support for the effectiveness of the NaP intervention.

Maternal depressive symptoms during pregnancy represent a significant risk for foetal development, newborn health, as well as for the infants’ later cognitive, socio-emotional and psychomotor development (Field, 2011; Kingston *et al.*, 2012; Field, 2017; Gentile, 2017). Prenatal symptoms are also likely to persist throughout the first postpartum year (Beeghly *et al.*, 2002; Austin *et al.*, 2007), with a potential to interfere with optimal child development, often through their negative impact on the early mother–infant interaction (Field, 2011).

Early interventions have predominantly focused on reducing mothers’ pre- and postnatal depressive symptoms based on the assumption that reducing depression would decrease its harmful consequences on parenting (Lefkovics *et al.*, 2014; Tsivos *et al.*, 2015; Field, 2017). Yet, reducing mothers’ depressive symptoms alone does not appear to lead to improvements in parenting or in infants’ well-being and development (Forman *et al.*, 2007; Nylen *et al.*, 2006; Tsivos *et al.*, 2015). Subsequently, it has been suggested that early interventions should also focus directly on enhancing optimal mother–infant relationships already during pregnancy and beyond and be offered in primary health care settings to increase their accessibility (Lefkovics *et al.*, 2014). In the present paper, we first introduce the development of an intervention model for mothers with depressive symptoms, the *Nurture and Play* (*NaP*), designed to enhance early maternal caregiving qualities starting from pregnancy and continuing to infant age of seven months. Second, we describe the results of a pilot RCT study, evaluating the effectiveness of NaP in decreasing maternal depressive symptoms, as well as enhancing maternal reflective functioning (RF) and emotional availability (EA) in the mother–child relationship.

The EA perspective suggests that at the core of a healthy mother–child relationship is the maternal capacity to read and respond to the infant’s emotional cues and the child’s reciprocity of emotional responding (Biringen and Easterbrooks, 2012; Biringen *et al.*, 2014). Maternal EA is a multidimensional construct, comprising dimensions of maternal *sensitivity*, that is, appropriate affective and behavioural responsiveness towards the child; *structuring*, that is, her ability to guide, teach and set limits while remaining in contact; *non-hostilit*y, that is, good regulation of negative affect and *non-intrusiveness*, that is, ability to follow child lead and to refrain from interfering behaviour towards him/her. From the child’s side, *responsiveness*, that is, appropriate affective responding towards the adult, and *involvement*, that is, actively seeking emotional contact with the adult, can be observed. We have shown in our previous study that some of the components of EA are already observable in maternal interactions towards her unborn child: Maternal sensitivity can be observed in her willingness to communicate with the foetus baby with positive emotions, and maternal non-hostility can be observed in the lack of hostile affects and behaviours towards the foetus baby (Salo *et al.*, 2019). Previously, these kinds of responses have also been described as parts of intuitive parenting, referring to the biological predisposition for human parenting (Papoušek and Papoušek, 2002).

With regard to prenatal depressive symptoms, some research shows that mothers with prenatal depressive symptoms display lower levels of emotional attachment towards the foetus (Alhusen, 2008; Yarcheski *et al.*, 2009). Furthermore, early postnatal mother–infant interactions are crucial for infant well-being and development, as infants are dependent on the caregiver’s aid in their emotion and stress regulation and cognitive learning (Sroufe, 2000; Calkins and Hill 2007). Infants typically tune their emotional signals towards the mother’s voice, gestures, movements and facial expressions (Trevarthen, 1998). Maternal depression can thus influence infant’s affective states and emotional responses (Tronick and Reck, 2009). Depressive mothers typically show flat and unexpressive facial expressions with the infant, and ample evidence shows low levels of EA among mothers with postpartum depression (Easterbrooks *et al.*, 2000; Van Doesum *et al.*, 2007). Research also indicates the protective influence of sensitive and positive dyadic interactions, even against a backdrop of maternal depression. Hayes *et al.* (2013) found that severe prenatal maternal depressive symptoms did not predict infant’s disorganised attachment at 12 months, if the postnatal mother–infant interaction involved more optimal levels of maternal warmth and positive regard. Likewise, maternal postnatal major depression did not increase infant’s problematic social engagement at nine months, if mothers showed high sensitivity in the dyadic interaction (Feldman *et al.*, 2009). Accordingly, the treatment elements in the NaP were designed to enhance both pre- and postnatal EA.

Another parenting variable relevant for healthy child development when mother has prenatal depressive symptoms may involve her RF, referring to the parental capability of explicitly describing feelings, intentions and thoughts underlying their own and others’ behaviour (Fonagy *et al.*, 1991; Slade *et al.*, 2009). Pregnancy is a unique phase that requires the mother to imagine the future and extend her current understanding of herself, her spouse and life situation to include the child (Slade *et al.*, 2009). Prenatal RF serves preparation to motherhood and some research shows that high prenatal RF, characterised by the capability of imagining oneself and the future child positively, predicts high quality of parent–infant interaction (Smaling *et al.*, 2016). In the postpartum period, parental RF is crucial for sensitive caretaking, as it fuels the mother’s accurate understanding of the intentional states of her infant and accurate interpretation of infant distress cues (Slade *et al.*, 2005). In a recent review, high levels of parental postnatal RF were shown to be systematically associated with children’s optimal socio-emotional development (Camoirano, 2017). Depressive individuals, on the other hand, tend to show low levels of symbolisation and willingness to explore their own inner mental state (Luyten *et al.*, 2012), suggestive of low RF (Slade *et al.*, 2005). Previous studies on depression and parental RF have so far only been conducted among high-risk substance-using mothers. A study in an intervention setting found that among mothers with substance use disorders, low level of RF was related to higher depression (Suchman *et al.*, 2010b), while another study in a residential treatment setting among substance-using women did not find association between maternal RF and psychiatric symptoms (Pajulo *et al.*, 2012). Nevertheless, it has been suggested that parental RF may aid the quality of dyadic interaction, especially in times of heightened distress as it allows mothers to step back from their immediate affective experience and have the capacity to reflect on their child’s internal experience (Fonagy *et al.*,^1991^; Slade *et al.*, 2005). Therefore, in NaP, we focused directly on improving the maternal skills for RF starting already from pregnancy.

Several programs aiming to prevent and/or reduce maternal depressive symptoms have been described in the literature. Effective treatment models include interpersonal and cognitive therapies as well as focused prevention programs involving a combination of psychoeducation about infant development and attachment and ways to strengthen peer social support (Elliott *et al.*, 2000; Zlotnick *et al.*, 2001; Zlotnick *et al.*, 2006; Claridge, 2014; Field, 2017). Further, yoga-based treatment (Narendran *et al.*, 2005; Field *et al.*, 2013) and massage therapy (Field *et al.*, 2010) have also been found effective.

However, interventions focusing on maternal mood disorders alone have not been found to be sufficient to buffer infant development or mother–infant interaction quality (Nylen *et al.*, 2006; Forman *et al.*, 2007; Poobalan *et al.*, 2007; O’Hara, 2009; Tsivos *et al.*, 2015). Even though maternal mood may improve, the negative effect of maternal mood disorders on parent–child interaction may continue to be manifest. For example, adult-focused psychotherapy has been found to be ineffective in sufficiently increasing maternal responsiveness or in improving child well-being from 6 to 18 months (Forman *et al.*, 2007). Only a few intervention studies have focused on intervention models with direct parenting components supplementing the treatment of mood disorders (Tsivos *et al.*, 2015), and even fewer begin during pregnancy (see Field, 2017).

When intervention studies have focused only on the postpartum period, the results are mixed. For example, van Doesum *et al.*, (2008) examined the effects of a mother–child intervention on the quality of mother–child interaction, infant–mother attachment security and infant socio-emotional functioning in dyads of depressed mothers when infants were 1–12 months old. The results showed that the intervention was effective in increasing the quality of mother–infant interaction. In contrast, a study using perinatal dyadic psychotherapy (PDP), involving both the mother and the infant, did not show changes in parent–child interaction (Goodman *et al.*, 2015).

Taken together, there are limitations in the above-mentioned intervention studies. First, most focused solely on the reduction of depressive symptoms instead of also enhancing parenting. Second, with few exceptions, the interventions with a dual focus on parenting and depressive symptoms started only during the postpartum period, although it is pregnancy that is agreed to be the crucial period for later maternal mental health, parenting and child development (Field, 2017). Third, there are mixed findings regarding effectiveness of parenting interventions in the postpartum period. The present study introduces a prenatal parenting intervention, the NaP, which can be offered to all new expectant mothers with depressive symptoms with the potential to reach a wide population of pregnant women. The program aims to reduce the negative impact of depressive symptoms on the mothers, the infants and their dyadic interaction and to strengthen maternal RF to enhance future mental health and optimal emotional interaction. First, to maximise the potential for population uptake, the NaP was developed to be a short-term, manualised and easily taught, group-based intervention that requires minimal training of the instructors and can be implemented by multiprofessional primary health care professionals (e.g., psychologist, well-baby clinic nurse, family worker) working in primary health care settings. Second, the program is introduced during pregnancy and continues until the infant is seven months old. By then, centering around the ages between three and six months, the most crucial phase in the development of bio-behavioural synchrony in dyadic interaction is over (Feldman, 2007). Also, as the NaP groups involve structured activities and reflective practices, for practical reasons, the set-up is easier to manage before the age of around seven months, when most infants will move, crawl and require a different intervention set-up suitable for their developing motor skills. Third, the NaP focuses directly on increasing maternal RF and EA in mother–infant interaction, thus strengthening the protective processes for infant development. Fourth, the intervention facilitates recovery from depressive symptoms by training the women through cognitive and affect regulation strategies, offering resources and peer support and fostering access to help and continuity of care into the transition to parenthood.

## Study aims and hypotheses

The present, randomised control study examines the effectiveness of the NaP intervention in increasing EA and maternal RF and reducing maternal depressive symptoms in a follow-up design from pregnancy to one year. The hypotheses are, first, that the EA (i.e., maternal sensitivity, structuring, non-intrusiveness, non-hostility and infant responsiveness and involvement) and RF at the age of 12 months are higher and depressive symptoms lower in the intervention group as compared to the treatment as usual (TAU) group. Second, we examined change and hypothesise that the two EA dimensions observable already during pregnancy (i.e., maternal sensitivity and non-hostility) and maternal RF will increase and, third, that maternal depressive symptoms will decrease from pregnancy to one year more in the mothers in the intervention group than in the control group (i.e., an interaction effect).

## Method

### Participants

The sample consisted of 45 women invited to the project by their well-baby clinic nurse. The invitation was made personally during a regular check-up if their Edinburgh Postnatal Depression Scale (EPDS; Murray and Cox, 1990) scores were 9 or higher and they were between 22 and 31 gestational weeks. The recruitments took place in four well-baby clinics in Lahti, a Southern town in Finland. If the mothers’ scores were beyond 13, they were also guided to appropriate communal adult psychiatric services unless they already had such a contact. Four mothers already had individual psychiatric contact during the project (one intervention and three control group mothers). All parents gave their voluntary, informed consent for treatment and were informed of their rights to leave the treatment at any time. The authors assert that all procedures contributing to this work comply with the ethical standards of the relevant national and institutional guidelines on human experimentation and with the Helsinki Declaration of 1975, as revised in 2008. The ethical committee of the City of Lahti approved the study plan.

All mothers meeting the inclusion criteria (*n* = 51) were invited to participate in the study, the enrolment time spanning one year (4/2012 and 5/2013). The agreement rate was 92%. Non-participation was due to practical issues related to childcare with older siblings, marital problems and lack of motivation. The fathers – if available – were invited to the first meeting and home visit after birth. Although they did not take part in the study or the intervention (for intervention group), they were given information about the study aims and the possibility of getting counselling help himself if they needed. All mothers in Finland receive bi-monthly medical check-ups during pregnancy and six meetings after the birth until the children are 12 months (Hakulinen-Viitanen and Pelkonen, 2009), so both intervention and TAU groups received these services. These routine check-ups last about 15 min, and the main focus is on the physical well-being of the mother and the child as well as giving general psychoeduation on maternal, paternal and infant psychosocial well-being and infant development (Hakulinen-Viitanen and Pelkonen, 2009). The control group mothers did not have any parenting-based intervention; they also did not have a special focus on the improvement of depressive symptoms. The authors of the present study did the scoring and reporting of the results. We were not involved in the data collection.

### Procedures

**Figure 1.**
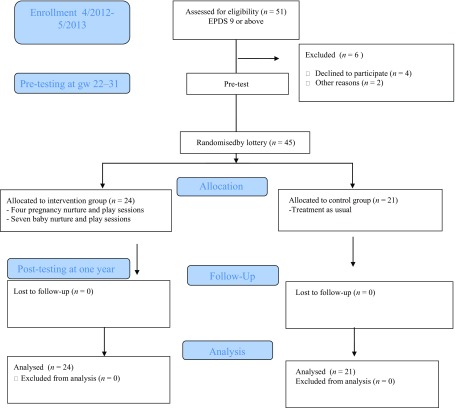
The design of the randomised control trial (RCT).

The design of the randomised control trial (RCT) is shown in the CONSORT (Moher *et al.*, 2010) diagram in Figure 1. Before randomisation, all 45 mothers were seen twice during pregnancy. This served as a baseline study phase, and data were collected on the study variables, and various background factors using interviews, observational measures and standardised questionnaires. After random selection (by lottery), 24 mothers were invited to participate in the NaP group intervention, while the rest of mothers (*n* = 21) served as a control group.

#### Nurture and play intervention

Table 1 presents the structure and treatment elements of each session in relation to the three goals of NaP intervention (Salo and Lampi, 2019). After meeting all the mothers individually (or with the father) and doing the interviews (which also served as the pre-test phase for the present study), the bi-weekly held Pregnancy Groups started. The Baby Groups were held weekly, with possible breaks during holidays. Each NaP session lasted 1.5 h. Afterwards, there were coffee/tea servings for the group for additional 30–45 min. The physical setting was made as comfortable as possible, with cushions, bean bag chairs and so on offered for seating. The Baby Group was invited to come 15–20 min prior to the starting to give mothers time for feeding, changing nappies and settling together with their baby to the group. First, *EA* was targeted by using playful, Theraplay-based activities (Booth and Jernberg, 2010), such as singing, playing musical instrument to the foetus or a postpartum, playing interactional activities such as infant massage and peek-a-boo with the infants. Theraplay is an active, adult-led, playful parent–child interaction therapy. All the Theraplay-based activities are designed to promote affectionate contact through physical touch and reciprocity (Pregnancy Groups) and synchrony and joint attention (Baby Groups). Depressed mothers have been shown to have specific problems in sensitive attunement, using less physical touch and in a less affectionate manner, and using of more negative vocal behaviour and less-infant directed speech (Field, 2010; 2011). In the Pregnancy NaP sessions, mothers were invited to touch their tummies, make rhythmic movements to stimulate the foetus and to stroke their tummies along with singing lullabies. In the Baby NaP sessions, the focus was on infant massage, various songs using rhythmic movements and holding and rocking the infant.

**Table 1.** Structure and examples of treatment elements of pregnancy and Baby NaP groups according to the intervention goals

	Emotional availability	Reflective functioning	Depressive symptoms
*Pregnancy Nurture and Play group*			
Session 1			
Mother’s attachment history	Writing to a diary: What kind of nurture I want to give to my baby?	Understanding links between past and present relationships	Psychoeducation on affect regulation
		Experience of being pregnant	
Session 2			
Connecting with the baby	Singing, exploring the babies’ senses with touch, light and music, for example, playing a musical instrument to the baby and following baby’s responses	Representations of the baby (e.g., drawing a picture of oneself and the baby)	Psychoeduation on depressive thoughts/how to deal with them
		Expectations of the baby	
Session 3			
Becoming a mother	Singing, exploring the babies senses and reactions	Representations of becoming a mother (using baby diary, selecting adjectives, etc.)	Mapping social support systems (homework for fathers)
	Relaxation techniques		
		Role transitions Ambivalence	
Session 4			
Preparing for birth	Drawing a card to the baby	Prepare for the birth and actual meeting with the baby	Dealing with practical issues related to sleep, possible fear of child-birth, social support for birth, etc.
	Relaxation techniques		
*Baby nurture and play groups*			
Sessions 1–3			
Getting to know the baby	Singing and playing with the babies	Reflecting on the birth experience, first weeks with baby (feelings, thoughts) and the current perceptions of the baby in the session	Checking mood using screeners
			Checking affect and thought regulation strategies
	Measuring, peek-a-boo, infant massage, lullaby’s		
Sessions 4–5			
Encouraging mutuality	Singing and playing with the babies	Relationship with the baby	Checking affect and thought regulation strategies
		Grounding mentalising to real observations in the sessions (using homework and activities (e.g., observe your child for 3 min, then tell what you thought the child is feeling, thinking)	
	Measuring, peek-a-boo, infant massage, lullaby’s		
	Taking care of the infant (nurturing)		
Sessions 6–7			
Preparing for the future	Singing and playing with the babies	Babies unique personalities	Checking affect and thought regulation strategies
		Taking developmental perspective; when babies start to separate	
			Social support systems
	Measuring, peek-a-boo, infant massage, lullaby’s, rough- and tumble play, soap bubbles, etc.	Representations of the future	

Second, in *terms of the RF*, each session dealt with a discussion topic that was chosen to activate the reflectiveness of the mother, including feelings and thoughts related to the pregnancy, maternal representations of their childhood history, their ideas about the child, their experience of being a mother and their hopes about their own as well as their child’s future. Here, the focus was on enhancing explicit parental RF (i.e., mentalisation about understanding how one’s own mental states such as feelings and thoughts influence interactive behaviour). In the Baby NaP Groups, the additional focus was also on how the child’s mental states operate and influence the child’s behaviour, as well as one’s own mind and behaviour. The most common techniques in mentalisation-based interventions were utilised to make the patterns of mother–child interaction more understandable (Midgley and Vrouva, 2013): These include *pausing technique*, focusing on the here and now, *active and explicit acknowledging* of feelings and thoughts and *stopping non-mentalising*.

Third, each session included various *cognitive and affect regulation techniques* with direct attention on handling the current depressive mood and related somatosensory experiences, such as sleep and eating patterns. Cognitive Behaviour Therapy (CBT) approaches have been found to reduce relapse rates after treatment for depression (Hollon *et al.*, 2006). They have also been found to be effective with pre- and postpartum depression (Chabrol *et al.*, 2002; Murray *et al.*, 2003). Thus, both identifying and modifying the frequency of helpful and harmful thoughts and increasing pleasant activities and social skills training were included. Coping strategies for handling stress were also actively practiced and shared between the group members during the sessions. In between the sessions, homework and diaries were used to both stimulate thinking and feeling towards the child as well as to find ways of coping with challenges in affect regulation. Relaxation techniques and massage practices were also offered to all mothers in line with previous research indications that such practices have been found to decrease prenatal depression (Field, 2017).

### Measures

#### Edinburg Postnatal Depression Scale

The EPDS is a widely used and reliable 10-item self-report for the assessment of symptoms of depression (Murray and Cox, 1990). It asks about feelings of happiness and sadness, fears, self-blame, sleeping problems and thoughts about harming oneself during the previous week on a four-point scale (0–3). It is commonly used for both pre-and postpartum to screen for depression (Venkatesh *et al.*, 2016). The cut-off for the presence of mild depressive symptoms is 9/10 (Murray and Cox, 1990). A cut-off of a 9 in the second and third trimester has been shown to have most predictive value for screening clinical depression during pregnancy (Bergink *et al.*, 2011). In the present study, a cut-off of 9 points was used. Cronbach’s alphas were 0.85 and 0.80 (pre- and postnatal, respectively).

#### Emotional availability: prenatal and infancy versions

Observations of EA were conducted on semi-structured videotaped setting designed by Ann Jernberg and her colleagues called the Marschak Interaction Method (MIM) (Jernberg *et al.*, 1985; Salo and Booth, 2019). In the MIM’s, mother is asked to perform playful activities with the foetus (Prenatal), for example, play a musical instrument to your tummy baby, or the baby (Infancy), or play a familiar game together (Infancy) (Salo and Booth, 2019). In practice, the mother is asked to read the MIM tasks from a set of cards that the experimenter gives. The materials needed are in small bags. Experimenter leaves the room. The videotaped situation lasts about 15–20 min.

EA scales (Biringen, 2008) were used to score the observations from the MIM setting. In the 4th edition of the original scales, EA is comprises of six dimensions – parental sensitivity, structuring, non-intrusiveness, non-hostility, child responsiveness and child involvement – rated on a seven-point Likert-type scales. All of the dimensions are rated as global perceptions (a Likert scale from 1 to 7) with the overall aim of capturing the emotional connection in the dyad. In the prenatal phase, adaptations of the two adult scales – sensitivity and non-hostility – were created to measure EA during pregnancy in collaboration with Z. Biringen (Salo *et al.*, 2016) with established validity with widely used early parenting measures (Salo *et al.*, 2019). Thus, prenatal sensitivity comprises the assessment of overall affective quality, attunement towards the foetus, evidenced, for example, by touching the tummy and commenting on the baby’s movements, and responding to them with positive affect, for example, being rated highly sensitive (7) would require expressions of positive affect in the face as well as gentle touching of the tummy, using hands to hold the tummy, stroking the tummy, turning head towards the tummy while talking to the foetus as opposed to a very still-face expression, not touching one’s tummy at all while performing the tasks, etc. In lower scores, there is either a pseudo-quality in maternal affect, that is, it is overly positive and bright and lacks authencity (4), or depressed and withdrawn affects with little orientation (psychological or behavioural) towards the foetus (3). In the lowest end of scores (1 and 2), there are awkward expressions, or total shutting down. Adult non-hostility characterises the ability to handle one’s negative emotions. It is manifested in the absence of hostile responses, and overt or covert hostile behaviour. The most hostile adult is openly exhibiting his or her hostility in facial expression and voice. Hidden or covert hostility includes showing impatience or boredom, but it need not be directed at the child. The high points refer to lack of any hostile qualities (7). The midpoint scores refer to covert hostility (4) where mother has occasionally negative expressions in face, posture and touch (tensed eyebrows, angry mouth, etc.). In lower scores, there are some to several expressions of hostility, for example, negativity in the face, posture or touch (such as poking the foetus), critical remarks, minimising the situation or the foetus, making sarcastic or negative comments warranting scores 3, 2 or 1, respectively.

A trained rater (first author) rated all the tapes, with 20% of the tapes rated also by a second trained rater (second author). Both were reliable in the EAS 4th edition and trained by Z. Biringen. Five tapes were checked together with the method developer (Z.B). Inter-rater reliability (Pearson’s *r*) was 0.89 for sensitivity and 0.84 for non-hostility, respectively, for the Prenatal EA and 0.92 for sensitivity, 0.88 for structuring, 0.79 for non-intrusiveness, 0.82 for non-hostility, 0.84 for child responsiveness and 0.80 for child involvement for the Infancy EA.

#### The pregnancy and parent development interviews

The Pregnancy Interview (PI-RF; Slade *et al.*, 2007) is a semi-structured clinical interview with 22 questions regarding a variety of mental states related to mothers’ emotional experience with pregnancy and her expectations, hopes and fears regarding her future relationship with the child (e.g., ‘What do you think will be the hardest times during the first six months of your baby’s life?’). The Parent Development Interview (PDI-RF; Slade *et al.*, 2005) is a 45 item semi-structured clinical interview intended to examine the parents’ representations of their children, themselves as parents and their relationships with the child. The interview strives in a number of ways to tap into parents’ understanding of their child’s behaviour, thoughts and feelings and asks the parents to provide real-life examples of charged interpersonal moments: ‘Describe a time in the last week when you and your child really clicked’ and then ‘a time when you and your child really didn’t click’.

In evaluating both pre- and postnatal RF, audiotaped narratives were transcribed verbatim and scored for parental RF by trained coders who were reliable in PI and PDI scoring by Arietta Slade or her team members (first and fifth author). Here, Finnish translations of the scoring manual were used (Pajulo, 2004). Both interviews take approximately 1–1.5 h to administer. The signs of mentalising coded from the interviews can be divided into four categories: (a) the parent’s awareness of the nature of different mental states, (b) the parent’s clear and exact intention to understand the mental states underlying behaviour, (c) the parent’s ability to recognise developmental aspect of mental states and d) the parent’s ability to consider mental states in relation to the interviewer. Freshness and spontaneity of reflections about specific interaction episodes are taken into account, and the importance of episodic memory is emphasised. Generalised expressions, opinions or clichés are not considered signs of true RF. The number of indications of true reflectiveness found in the transcribed narrative is the basis for assigning an overall score. The greater the number of specific and varied indications of RF, the higher the score on an eleven-point scale, with a score of −1 indicating a rejection of RF and scores 6–9 representing exceptionally high ability for RF. Twenty percent of the interviews were scored by two independent raters (first and fifth author), and the inter-rater-reliability was 0.95 (Pearson’s *r*) for the PI and 0.98 (Pearson’s *r*) for the PDI.

### Analysis strategy

Missing values were replaced with expectation-maximisation (EM). To test for the success of the randomisation procedure, group differences in background variables (educational level, marital status and parity) were tested with Fisher’s exact tests and pre-intervention levels of study variables (mother’s sensitivity, non-hostility, RF and depressive symptoms) with Student’s *t*-tests. Student’s *t*-tests were conducted to address the first research question, whether mother–infant EA (i.e., maternal sensitivity, structuring, non-intrusiveness, non-hostility and infant responsiveness and involvement) and maternal RF at the age of 12 months are higher and maternal depressive symptoms lower in the intervention group as compared to the TAU group. To address the second research question, whether the two EA dimensions observable already during pregnancy (i.e., maternal sensitivity and non-hostility), and maternal RF will increase and third that maternal depressive symptoms will decrease from pregnancy to one year more in the mothers in the intervention group than in the TAU group (i.e., a interaction effect), three repeated measure analyses of variances (ANOVAs) were conducted. The intervention/TAU group was the independent variable and pre- and post-intervention measurements of (a) EA maternal sensitivity and non-hostility towards the infant, (b) maternal RF and (c) maternal depressive symptoms were the dependent time variables.

## Results

### Descriptive statistics

Background characteristics for the intervention and TAU group are shown in Table 2. Socioeconomic status was assessed by the level of education from 1 (primary school), 2 (high school and trade school), 3 (university degree) and 4 (doctoral degree). Due to sample size, it was re-classified into two classes, representing low (primary and high or trade school) and high educational level (university and doctoral degrees). Over half had either high school or trade school or university degree of education. Most mothers were married or co-habiting, and over half were first-time mothers. The groups did not differ in any of the background characteristics.

**Table 2.** Percentage (%) distributions of the background characteristics by group status (intervention versus comparison)

	Intervention		Control			
	*n*	*%*	*n*	*%*	*Fisher’s* exact test *P*	
Educational level					0.22	
Low	12	50	15	71.4		
High	12	50	6	28.6		
Marital status					0.11	
Married or co-habiting	19	82.6	21	100		
Single	4	17.4	0	0		
Parity					0.07	
Primiparous	13	54.2	17	81		
Multiparous	11	45.8	4	19		

To test the success of the randomisation procedure, we tested the differences in group means in study variables at baseline, shown in Table 3. No group differences were found.

**Table 3.** Mean and standard deviations of the baseline variables in intervention and control groups

	Intervention		Control			
	*M*	*SD*	*M*	*SD*	*t (43)*	*P*
Pre-intervention RF	3.05	1.34	2.65	1.44	−0.96	0.34
Pre-intervention sensitivity	3.54	1.21	3.38	1.08	−0.46	0.65
Pre-intervention non-hostility	4.03	1.03	3.65	1.17	−1.14	0.26
Pre-intervention depression	12.67	3.02	11.10	3.48	−1.62	0.11

Our first question was whether mother–infant EA (i.e., maternal sensitivity, structuring, non-intrusiveness, non-hostility and infant responsiveness and involvement) and maternal RF at the age of 12 months were higher and maternal depressive symptoms lower in the intervention group as compared to the TAU group. In line with the hypothesis, results in Table 4 show that maternal RF and sensitivity were higher among intervention than TAU group mothers. However, contrary to the hypotheses, there were no group differences in maternal structuring, maternal non-intrusiveness, maternal non-hostility, infant responsiveness, infant involvement or maternal depressive symptoms.

**Table 4.** Group differences in maternal RF, EA and depressive symptoms at child age of one year (post-intervention)

	Intervention		Control			
	*M*	*SD*	*M*	*SD*	*t (43)*	*P*
Maternal RF	4.05	1.33	2.92	1.45	−2.73	**0.009**
Maternal sensitivity	4.77	0.68	3.76	0.96	−4.13	**<0.001**
Maternal structuring	4.07	0.93	4.10	0.94	0.11	0.91
Maternal non-intrusiveness	4.57	1.53	4.45	1.53	−0.31	0.76
Maternal non-hostility	4.56	0.77	4.47	1.06	−0.33	0.74
Child responsiveness	4.27	0.74	4.13	1.04	−0.52	0.60
Child involvement	3.72	0.80	3.89	0.91	0.68	0.50
Maternal depression	8.16	2.99	9.65	3.92	1.42	0.16

**Figure 2.**
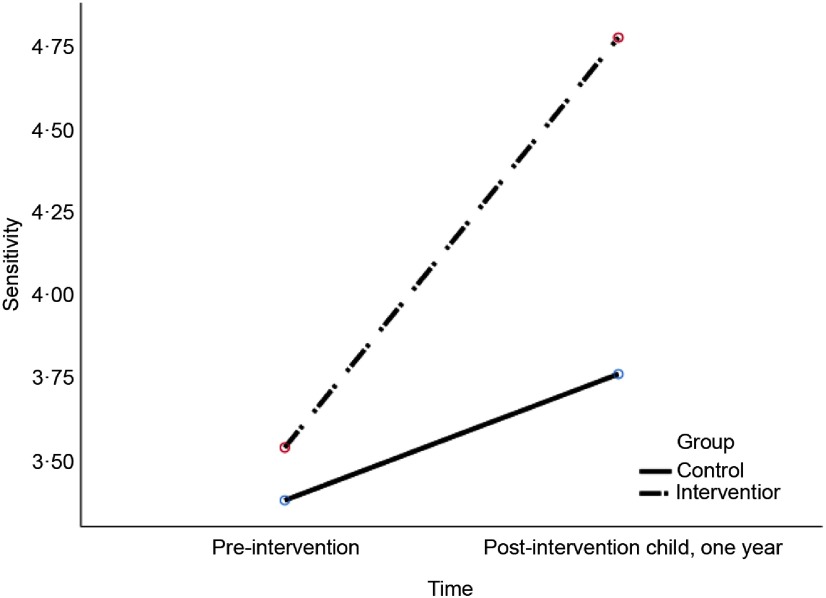
Change in maternal sensitivity from pre- to post-intervention

Our second question was whether intervention group mothers showed a greater increase in maternal sensitivity, non-hostility and RF and greater decrease in depressive symptoms from pregnancy (pre-intervention) to one year of age (post-intervention). Related to change in maternal EA variables from pre- to post-intervention, the results showed a significant group × time interaction effect, *F*_*Wilks’ Lambda*_ (2, 42) = 6.63, *P* = 0.003, *η* ^2^ = 0.24. In accordance with the hypothesis, univariate tests confirmed that mothers in the intervention group showed a greater increase in EA sensitivity than control mothers, *F* (1, 43) = 9.26, *P* = 0.004, *η* ^2^ = 0.18, illustrated in Figure 2. However, contrary to the first hypothesis, the intervention did not improve maternal non-hostility, *F*(1, 43) = 0.62, *P* = 0.44, *η* ^2^ = 0.014. Significant main effects showed that both groups showed increased sensitivity and non-hostility from pre- to post-intervention, *F*_*Wilks’*_ _*Lambda*_(2, 42) = 17.64, *P* < 0.001, *η* ^2^ = 0.46. Univariate tests indicated that the effect was significant both for sensitivity, *F*(1, 43) = 33.07, *P* < 0.001, *η* ^2^ = 0.44, and non-hostility, *F*(1, 43) = 14.23, *P* < 0.001, *η* ^2^ = 0.25.

**Figure 3.**
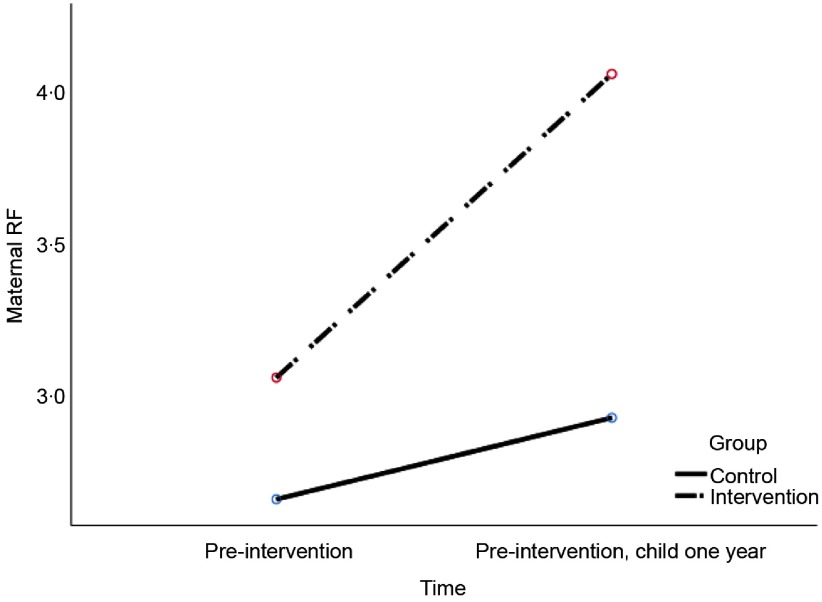
Change in maternal reflective functioning from pre- to post-intervention

Related to change in maternal RF from pre- to post-intervention, the results showed a significant group × time interaction effect, *F*_*Wilks’ Lambda*_ (1, 43) = 9.49, *P* = 0.004, *η* ^2^ = 0.18. Figure 3 shows, in accordance with the hypothesis, that intervention group mothers showed a greater increase in RF than control mothers. Yet, significant main effects indicate all mothers showed increase in RF from pre- to post-intervention, *F*_*Wilks’ Lambda*_ (1, 43) = 28.53, *P* < 0.001, *η* ^2^ = 0.40.

**Figure 4.**
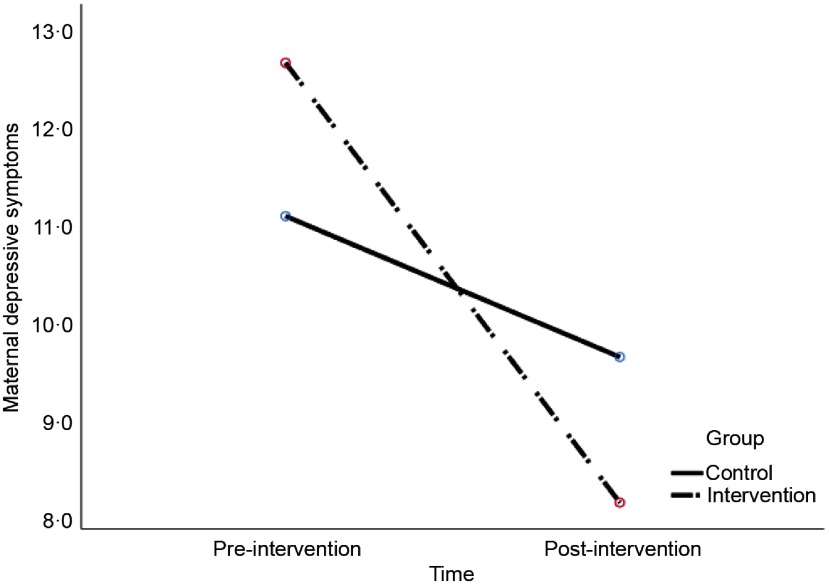
Change in maternal depressive symptoms from pre- to post-intervention

Related to change in mother’s depressive symptoms from pre- to post-intervention, the results showed a significant group × time interaction effect, *F*_*Wilks’ Lambda*_ (1, 43) = 5.40, *P* = 0.025, *η* ^2^ = 0.11. Figure 4 shows, as hypothesised, that intervention group mothers showed a greater decrease in depressive symptoms than the control mothers. Yet, all mothers showed decrease in depressive symptoms from pre- to post intervention, *F*_*Wilks’ Lambda*_ (1, 43) = 20.38, *P* < 0.001, *η* ^2^ = 0.3.

## Discussion

NaP is a mentalisation-based parenting intervention aimed to support mother–infant interaction quality among prenatally depressed mothers in primary health care settings. This study showed preliminary support for the effectiveness of NaP intervention model for mothers with prenatal depressive symptoms. Specifically, the results supported the hypotheses that maternal EA dimension of sensitivity as well as maternal RF was higher at one year of child age in the NaP intervention group as compared to the TAU group. Also, the results further showed that maternal sensitivity and RF increased, whereas depressive symptoms decreased more in the intervention group from pre- to postnatal phase although in both groups there were positive changes.

Maternal sensitivity is viewed as a key characteristic underlying the development of a child’s secure attachment (De Wolff and Van Ijzendoorn, 1997; Ainsworth *et al.*, 2015). It encompasses the core sense of safety that another human being is willing and able to fully read, understand and respond to one’s needs, which in infancy means survival. In the EA framework, a sensitive mother expresses positive emotions towards the child and is open towards his/her communication cues and signals (Biringen *et al.*, 2014). Here, intervention group mothers showed more sensitivity when the baby was one year old, indicating the benefits of participating in the NaP intervention. Furthermore, although emotional sensitivity expressed towards the foetus in videotaped assessment is likely to be different than the reciprocal relational sensitivity with the infant, increased maternal sensitivity from pregnancy until the baby was one year old indicates that it is possible to encourage depressive mothers to develop a positive emotional bond already from pregnancy. The result of the effectiveness of the NaP in increasing maternal sensitivity from pre-to postnatal period is important, because depressive mothers tend to have negative feelings already towards the foetus (Alhusen, 2008). Such NaP therapeutic elements as rhythmic touch and emotion interpretation to help the pregnant mother to feel, focus and reciprocate the movements of the future baby may have contributed to the improvements in sensitivity.

Yet, in contrary to the hypotheses, the other dimensions of the EA – namely, structuring, non-intrusiveness, non-hostility and child responsiveness and involvement did not differ between the study groups. Furthermore, NaP was not effective in reducing maternal hostility from pre- to postnatal period. Mothers suffering from depressive symptoms often show hostile and intrusive interactive behaviours with their infants and face difficulties in guiding and structuring their interaction and communication (Field, 2010; Murray *et al.*, 2015). However, because the NaP intervention highly focused very much on creating positive emotional connection and pleasurable moments of meeting, it is possible that it neglected to focus on the other EA dimensions, here reducing hostility. In the present sample, the level of hostility was not as low as sensitivity. Nevertheless, in the future, it might be relevant to add interventive elements helping mothers to increasingly understand her problems in affect regulation and possible trauma history which is typical in maternal prenatal depression (Seng *et al.*, 2014) as these techniques have previously been found to be the effective in reducing hostility and intrusiveness (Belt *et al.*, 2012). In the future, it might be relevant to add interventive elements helping mothers to increasingly understand her problems in affect regulation and possible trauma history which is typical in maternal prenatal depression (Seng *et al.*, 2014) as these techniques have previously been found effective in reducing hostility and intrusiveness (Belt *et al.*, 2012). Similarly, the NaP did not reveal any effects on the infants’ side of the EA, namely, responsiveness and involvement. As maternal depressive symptoms have been show to have negative effects, especially involving the child’s emotional responsiveness and interactive behaviours (Field, 2011), focusing more directly on the infant’s side of the emotional interaction in future development of the NaP seems relevant.

The NaP intervention was effective in increasing maternal RF more among the intervention than control mothers. Interestingly also, RF increased in both intervention and TAU groups from pregnancy to infancy, but the improvement was significantly more substantial for those in the intervention group. This overall increase in RF from pre- to postnatal phase has been demonstrated previously in a non-randomised intervention study with substance-abusing mothers (Pajulo *et al.*, 2012). Pre- and postnatal RF are by nature qualitatively different: one measuring imaginary relationship and the other actual perceptions of the child and related mentalising of the child’s behaviours. Such improvements are nevertheless important, because the high capacity for RF is thought to be beneficial for dyadic relations and found to be a protective factor for infant development (Camoirano, 2017). The prior evidence of the effectiveness of early RF-based interventions focused on mothers with substance-use problems (Pajulo *et al.*, 2006; Suchman *et al.*, 2008; 2010a; 2010b; Pajulo *et al.*, 2012; Suchman *et al.*, 2017) as well as mothers living in the prison system (Baradon *et al.*, 2008). The current study extends the previous findings to mothers with prenatal depressive symptoms.

The finding that NaP intervention was also effective in reducing depressive symptoms concurs with previous findings on interventions using similar therapeutic elements (Elliott *et al.*, 2000; Zlotnick *et al.*, 2001; 2006; Field, 2010). Again, although depressive symptoms also decreased in the control group, the reduction was more pronounced in the intervention group. Previous research has shown that for some women, postpartum depression spontaneously remits. In Finland, the well-baby clinic provides regular medical check-ups during pregnancy and after birth, aiming to contribute to optimal infant development and maternal health, offering also information about depressed mood (Hakulinen-Viitanen and Pelkonen, 2009). Our finding that this TAU with depression monitoring by a public health nurse may be an effective intervention is supported by a number of studies which have shown that low-intensity interventions, such as guided self-help via written materials, limited professional support, or Internet-delivered interventions are effective in postpartum patients with Major Depressive Disorder, even when depression is severe (Bower *et al.*, 2013). They are, however, hardly effective on improving parenting problems inherent in maternal depression (see Lefkovics *et al.*, 2014).

The limitations of the study are that the sample size was small, and the results need to be verified using a larger sample. The use of multiple qualitative measurements including transcribed interviews and videotaped recordings may nevertheless pose practical challenges for larger intervention studies. Furthermore, as the effects of parental depressive problems in terms of the later child development and health are often cumulative in nature (Goodman *et al.*, 2011), the role of the fathers’ mental health, dyadic father–infant interaction and paternal reflective function need more attention. In the present study, fathers were offered additional help in guiding them into necessary mental health services if needed, but they did not take part in the group sessions

Despite these limitations, our study adds to the existing literature on early intervention programs for mothers with depressive tendencies starting from pregnancy. Pregnancy is a unique phase that requires the mother to envision many unknowns about her life situation, her spouse, and of course, the formation of an emotional bond to her child. NaP adds to the science of early interventions by focusing also on the positive aspects of early caregiving. NaP is a short-term, cost-effective group-based intervention that can be easily delivered multiprofessionally in primary health care. Thus, this study contributes to the discussion on the appropriate interventions for prenatal interventive work in at-risk samples, focusing especially on the early emotional signs of caregiving. Future studies with follow-up of the present sample will focus on evaluating the long-term effects of the NaP intervention.

## References

[ref1] Ainsworth MDS , Blehar MC , Waters E and Wall SN (2015) Patterns of attachment: A psychological study of the strange situation. New York: Psychology Press.

[ref2] Alhusen JL (2008) A literature update on maternal–foetal attachment. Journal of Obstetric, Gynecologic & Neonatal Nursing, 37, 315–328. 10.1111/j.1552-6909.2008.00241.x PMC3027206 18507602

[ref3] Austin MP , Tully L and Parker G (2007) Examining the relationship between antenatal anxiety and postnatal depression. Journal of Affective Disorders, 101, 169–174. 17196663 10.1016/j.jad.2006.11.015

[ref4] Baradon T , Fonagy P , Bland K , Lénárd K and Sleed M (2008) New beginnings–an experience-based programme addressing the attachment relationship between mothers and their babies in prisons. Journal of Child Psychotherapy, 34, 240–258.

[ref5] Beeghly M , Weinberg MK , Olson KL , Kernan H , Riley J and Tronick EZ (2002) Stability and change in level of maternal depressive symptomatology during the first postpartum year. Journal of Affective Disorders, 71, 169–180. 12167513 10.1016/s0165-0327(01)00409-8

[ref6] Belt RH , Flykt M , Punamäki RL , Pajulo M , Posa T and Tamminen T (2012) Psychotherapy groups and individual support to enhance mental health and early dyadic interaction among drug‐abusing mothers. Infant Mental Health Journal, 33, 520–534. 28520270 10.1002/imhj.21348

[ref7] Bergink V , Kooistra L , Lambregtse-van den Berg MP , Wijnen H , Bunevicius R , van Baar A and Pop V (2011) Validation of the Edinburgh depression scale during pregnancy. Journal of Psychosomatic Research, 70, 385–389. 21414460 10.1016/j.jpsychores.2010.07.008

[ref8] Biringen Z (2008). Emotional Availability (EA) scales manual, fourth edtion Part 1: Infancy/Early Childhood version (child aged 0–5 years). Unpublished manuscript. Boulder.

[ref9] Biringen Z , Derscheid D , Vliegen N , Closson L and Easterbrooks MA (2014) Emotional availability (EA): theoretical background, empirical research using the EA Scales, and clinical applications. Developmental Review, 34, 114–167.

[ref10] Biringen Z and Easterbrooks MA (2012) Emotional availability: concept, research, and window on developmental psychopathology. Development and Psychopathology, 24, 1–8. 22292989 10.1017/S0954579411000617

[ref11] Booth P and Jernberg AM (2010) Theraplay: helping parents and children build better relationships through attachment-based play. San Francisco, CA: Jossey-Bass.

[ref12] Bower P , Kontopantelis E , Sutton A , Kendrick T , Richards DA , Gilbody S , Knowles S , Cuijpers P , Andersson G , Christensen H and Meyer B (2013) Influence of initial severity of depression on effectiveness of low intensity interventions: meta-analysis of individual patient data. BMJ, 346–540. 10.1136/bmj.f540 PMC3582703 23444423

[ref13] Calkins SD and Hill A (2007) Caregiver influences on emerging emotion regulation In Gross JJ , editor, Handbook of emotion regulation. The Guilford Press: New York, 229–248.

[ref14] Camoirano A (2017) Mentalizing makes parenting work: a review about parental reflective functioning and clinical interventions to improve it. Frontiers in Psychology, 8, 14. 28163690 10.3389/fpsyg.2017.00014 PMC5247433

[ref15] Chabrol H , Teissedre F , Saint-Jean M , Teisseyre N , Roge B and Mullet E (2002) Prevention and treatment of post-partum depression: a controlled randomized study on women at risk. Psychological Medicine, 32, 1039–1047. 12214785 10.1017/s0033291702006062

[ref16] Claridge AM (2014) Efficacy of systemically oriented psychotherapies in the treatment of perinatal depression: a meta-analysis. Archives of Women’s Mental Health, 17, 3–15. 10.1007/s00737-013-0391-6 24240636

[ref17] De Wolff MS and Van Ijzendoorn MH (1997) Sensitivity and attachment: a meta‐analysis on parental antecedents of infant attachment. Child Development, 68, 571–591. 9306636

[ref18] Easterbrooks A , Biesecker G and Lyons-Ruth K (2000) Infancy predictors of emotional availability in middle childhood: the roles of attachment security and maternal depressive symptomatology. Attachment & Human Development, 2, 170–187. 11707909 10.1080/14616730050085545

[ref19] Elliott SA , Leverton TJ , Sanjack M , Turner H , Cowmeadow P , Hopkins J and Bushnell D (2000) Promoting mental health after childbirth: a controlled trial of primary prevention of postnatal depression. British Journal of Clinical Psychology, 39, 223–241. 11033746 10.1348/014466500163248

[ref20] Feldman R (2007) Parent–infant synchrony and the construction of shared timing; physiological precursors, developmental outcomes, and risk conditions. Journal of Child Psychology and Psychiatry, 48, 329–354. 17355401 10.1111/j.1469-7610.2006.01701.x

[ref21] Feldman R , Granat A , Pariente C , Kanety H , Kuint J and Gilboa-Schechtman E (2009) Maternal depression and anxiety across the postpartum year and infant social engagement, fear regulation, and stress reactivity. Journal of the American Academy of Child & Adolescent Psychiatry, 48, 919–927. 19625979 10.1097/CHI.0b013e3181b21651

[ref22] Field T (2010) Postpartum depression effects on early interactions, parenting, and safety practices: a review. Infant Behavior and Development, 33, 1–6. 19962196 10.1016/j.infbeh.2009.10.005 PMC2819576

[ref23] Field T (2011) Prenatal depression effects on early development: a review. Infant Behavior and Development , 34, 1–14. 20970195 10.1016/j.infbeh.2010.09.008

[ref24] Field T (2017) Prenatal depression risk factors, developmental effects and interventions: a review. Journal of Pregnancy and Child Health, 4, 301. 28702506 10.4172/2376-127X.1000301 PMC5502770

[ref25] Field T , Diego M , Delgado J and Medina L (2013) Yoga and social support reduce prenatal depression, anxiety and cortisol. Journal of Bodywork and Movement therapies, 17, 397–403. 24138994 10.1016/j.jbmt.2013.03.010

[ref26] Field T , Diego M and Hernandez-Reif M (2010) Prenatal depression effects and interventions: a review. Infant Behavior and Development, 33, 409–418. 20471091 10.1016/j.infbeh.2010.04.005 PMC2933409

[ref27] Fonagy P , Steele M , Steele H , Moran GS and Higgitt AC (1991) The capacity for understanding mental states: the reflective self in parent and child and its significance for security of attachment. Infant Mental Health Journal, 12, 201–218.

[ref28] Forman DR , O’Hara MW , Stuart S , Gorman LL , Larsen KE and Coy KC (2007) Effective treatment for postpartum depression is not sufficient to improve the developing mother–child relationship. Development and Psychopathology, 19, 585–602. 17459185 10.1017/S0954579407070289

[ref29] Gentile S (2017) Untreated depression during pregnancy: Short-and long-term effects in offspring. A systematic review. Neuroscience, 342, 154–166. 26343292 10.1016/j.neuroscience.2015.09.001

[ref31] Goodman JH , Prager J , Goldstein R and Freeman M (2015) Perinatal dyadic psychotherapy for postpartum depression: a randomized controlled pilot trial. Archives of Women’s Mental Health, 18, 493–506. 10.1007/s00737-014-0483-y PMC4439372 25522664

[ref32] Goodman SH , Rouse MH , Connell AM , Broth MR , Hall CM and Heyward D (2011) Maternal depression and child psychopathology: a meta-analytic review. Clinical Child and Family Psychology Review, 14, 1–27. 21052833 10.1007/s10567-010-0080-1

[ref33] Hakulinen-Viitanen T and Pelkonen M (2009) Lastenneuvola lapsen ja perheen terveyden ja hyvinvoinnin edistäjänä In Lammi-Taskula J , Karvonen S and Salme S , editors, Lapsiperheiden hyvinvointi. Helsinki: THL, 152–161.

[ref34] Hayes LJ , Goodman SH and Carlson E (2013) Maternal antenatal depression and infant disorganized attachment at 12 months. Attachment & Human Development, 15, 133–153. 23216358 10.1080/14616734.2013.743256 PMC3594350

[ref35] Hollon SD , Stewart MO and Strunk D (2006) Enduring effects for cognitive behaviour therapy in the treatment of depression and anxiety. Annual Review of Psychology, 57, 285–315. 10.1146/annurev.psych.57.102904.190044 16318597

[ref37] Jernberg A , Wickersham M and Thomas E (1985) Prenatal MIM. Unpublished Handbook. Chicago: The Theraplay Institute.

[ref38] Kingston D , Tough S and Whitfield H (2012) Prenatal and postpartum maternal psychological distress and infant development: a systematic review. Child Psychiatry & Human Development, 43, 683–714. 22407278 10.1007/s10578-012-0291-4

[ref39] Lefkovics E , Baji I and Rigó J (2014) Impact of maternal depression on pregnancies and on early attachment. Infant Mental Health Journal, 35, 354–365. 25798487 10.1002/imhj.21450

[ref40] Luyten P , Fonagy P , Lemma A and Target M (2012) Depression In Bateman AW and Fonagy P , editors, Handbook of mentalizing. Washington, DC: American Psychiatric Publishing, Inc., 385–418.

[ref41] Midgley N and Vrouva I , editors, (2013) Minding the child: Mentalization-based interventions with children, young people and their families. London: Routledge.

[ref42] Moher D , Hopewell S , Schulz KF , Montori V , Gotzsche PC , Devereaux PJ , Elbourne D , Egger M , & Altman DG (2010) CONSORT 2010 statement: updated guidelines for reporting parallel group randomised trials. BMJ (Clinical Research Ed), 340, 332. 10.1136/bmj.c869 PMC2844943 20332511

[ref43] Murray D and Cox JL (1990) Screening for depression during pregnancy with the Edinburgh Depression Scale (EDDS). Journal of Reproductive and Infant Psychology, 8, 99–107.

[ref44] Murray L , Cooper PJ , Wilson A and Romaniuk H (2003) Controlled trial of the short-and long-term effect of psychological treatment of post-partum depression: 2. Impact on the mother–child relationship and child outcome. The British Journal of Psychiatry, 182, 420–427. 12724245

[ref45] Murray L , Fearon P and Cooper P (2015) Postnatal depression, mother–infant interactions, and child development In Gemmill JMAW , Fearon P , editors, Identifying perinatal depression and anxiety: Evidenced-based practice in screening, psychosocial assessment, and management. Wiley 139–164.

[ref46] Narendran S , Nagarathna R , Narendran V , Gunasheela S and Nagendra HRR (2005) Efficacy of yoga on pregnancy outcome. Journal of Alternative & Complementary Medicine, 11, 237–244. 15865489 10.1089/acm.2005.11.237

[ref47] Nylen KJ , Moran TE , Franklin CL and O’Hara MW (2006) Maternal depression: a review of relevant treatment approaches for mothers and infants. Infant Mental Health Journal: Official Publication of The World Association for Infant Mental Health, 27, 327–343. 10.1002/imhj.20095 28640416

[ref48] O’Hara MW (2009) Postpartum depression: what we know. Journal of Clinical Psychology, 65, 1258–1269. 19827112 10.1002/jclp.20644

[ref49] Pajulo M (2004) Finnish translations of the PI and PDI manuals. Unpublished manuscript. Turku: Turku University.

[ref50] Pajulo M , Pyykkönen N , Kalland M , Sinkkonen J , Helenius H , Punamäki RL and Suchman N (2012) Substance‐abusing mothers in residential treatment with their babies: importance of pre- and postnatal maternal reflective functioning. Infant Mental Health Journal, 33, 70–81. 22899872 10.1002/imhj.20342 PMC3418818

[ref51] Pajulo M , Suchman N , Kalland M and Mayes L (2006) Enhancing the effectiveness of residential treatment for substance abusing pregnant and parenting women: focus on maternal reflective functioning and mother–child relationship. Infant Mental Health Journal: Official Publication of The World Association for Infant Mental Health, 27, 448–465. 10.1002/imhj.20100 PMC2813060 20119507

[ref52] Papoušek H and Papoušek M (2002) Intuitive parenting In Bornstein MH , editor, Handbook of parenting Volume 2. Biology and ecology of parenting, 182. New Jersey: Lawrence Erlbaum Associates, 183–207.

[ref53] Poobalan AS , Aucott LS , Ross L , Smith WCS , Helms PJ and Williams JH (2007) Effects of treating postnatal depression on mother–infant interaction and child development: systematic review. The British Journal of Psychiatry, 191, 378–386. 17978316 10.1192/bjp.bp.106.032789

[ref54] Salo S and Booth P (2019) The MIM handbook. Chicago: Theraplay Institutute.

[ref55] Salo S , Flykt M and Biringen Z (2016) Pregnancy version of the Emotional Availability Scales (Pre-EA). Unpublished Manual. Helsinki: Helsinki University.

[ref56] Salo S , Flykt M , Isosävi S , Punamäki R-L , Kallnd M , Biringen Z and Pajulo M (2019) Validating an observational measure of prenatal emotional availability among mother’s with depressive tendencies. Journal of Prenatal and Perinatal Psychology and Health , 34, 1–23.

[ref57] Salo S and Lampi H (2019) Nurture and play intervention for groups manual. Unpublished Manual. Helsinki: Helsinki University.

[ref58] Seng JS , D’andrea W and Ford JD (2014) Complex mental health sequelae of psychological trauma among women in prenatal care. Psychological Trauma: Theory, Research, Practice, and Policy, 6, 41. 10.1037/a0031467 PMC4280853 25558308

[ref59] Slade A , Bernbach E , Grienenberger J , Levy D and Locker A (2005) Addendum to reflective functioning scoring manual for use with the Parent Development Interview. Unpublished Manuscript. New York: City University of New York.

[ref60] Slade A , Cohen LJ , Sadler LS and Miller M (2009) The psychology and psychopathology of pregnancy. Handbook of Infant Mental Health, 3, 22–39.

[ref61] Slade A , Grienenberger J , Bernbach E , Levy D and Locker A (2005) Maternal reflective functioning, attachment, and the transmission gap: a preliminary study. Attachment and Human Development , 7, 283–298. 16210240 10.1080/14616730500245880

[ref62] Slade A , Patterson M and Miller M (2007) Addendum to Fonagy, Target, Steele & Steele reflective functioning scoring manual for use with the Pregnancy Interview. Unpublished Manuscript. New York, NY: The City College and Graduate Center of the City University of New York.

[ref63] Smaling HJ , Huijbregts SC , Suurland J , Heijden KB , Mesman J , Goozen SH and Swaab H (2016) Prenatal reflective functioning and accumulated risk as predictors of maternal interactive behavior during free play, the still‐face paradigm, and two teaching tasks. Infancy, 21, 766–784.

[ref64] Sroufe LA (2000) Early relationships and the development of children. Infant Mental Health Journal, 21, 67–74.

[ref65] Suchman N , DeCoste C , Castiglioni N , Legow N and Mayes L (2008) The mothers and toddlers program: preliminary findings from an attachment-based parenting intervention for substance-abusing mothers. Psychoanalytic Psychology, 25, 499. 20057923 10.1037/0736-9735.25.3.499 PMC2802496

[ref66] Suchman NE , DeCoste C , Castiglioni N , McMahon TJ , Rounsaville B and Mayes L (2010a) The mothers and toddlers program, an attachment-based parenting intervention for substance using women: post-treatment results from a randomized clinical pilot. Attachment & Human Development, 12, 483–504. 20730641 10.1080/14616734.2010.501983 PMC2928150

[ref67] Suchman NE , DeCoste C , Leigh D and Borelli J (2010b) Reflective functioning in mothers with drug use disorders: implications for dyadic interactions with infants and toddlers. Attachment & Human Development, 12, 567–585. 20931415 10.1080/14616734.2010.501988 PMC2953729

[ref68] Suchman NE , DeCoste CL , McMahon TJ , Dalton R , Mayes LC and Borelli J (2017) Mothering from the inside out: results of a second randomized clinical trial testing a mentalization-based intervention for mothers in addiction treatment. Development and psychopathology, 29, 617–636. 28401850 10.1017/S0954579417000220 PMC5407293

[ref69] Trevarthen C (1998) The concept and foundations of infant intersubjectivity In Braten S , editor, Intersubjective communication and emotion in early ontogeny. Cambridge: University Press, 15–46.

[ref70] Tronick E and Reck C (2009) Infants of depressed mothers. Harvard Review of Psychiatry, 17, 147–156. 19373622 10.1080/10673220902899714

[ref71] Tsivos ZL , Calam R , Sanders MR and Wittkowski A (2015) Interventions for postnatal depression assessing the mother–infant relationship and child developmental outcomes: a systematic review. International Journal of Women’s Health, 7, 429. 10.2147/IJWH.S75311 PMC4412485 25960678

[ref72] Van Doesum K , Hosman M , Riksen-Walraven J and Hoefnagels C (2007) Correlates of depressed mothers’ sensitivity toward their infants: the role of maternal, child, and contextual characteristics. Journal of the American Academy of Child and Adolescent Psychiatry, 46, 747–756. 17513987 10.1097/CHI.0b013e318040b272

[ref73] Van Doesum KT , Riksen-Walraven JM , Hosman CM and Hoefnagels C (2008) A randomized controlled trial of a home‐visiting intervention aimed at preventing relationship problems in depressed mothers and their infants. Child Development, 79, 547–561. 18489412 10.1111/j.1467-8624.2008.01142.x

[ref74] Venkatesh KK , Nadel H , Blewett D , Freeman MP , Kaimal AJ and Riley LE (2016) Implementation of universal screening for depression during pregnancy: feasibility and impact on obstetric care. American Journal of Obstetrics and Gynecology, 215, 517–e1. 27210067 10.1016/j.ajog.2016.05.024

[ref75] Yarcheski A , Mahon NE , Yarcheski TJ , Hanks MM and Cannella BL (2009) A meta-analytic study of predictors of maternal–fetal attachment. International Journal of Nursing Studies, 46, 708–715. 19081091 10.1016/j.ijnurstu.2008.10.013

[ref76] Zlotnick C , Johnson SL , Miller IW , Pearlstein T and Howard M (2001) Postpartum depression in women receiving public assistance: pilot study of an interpersonal-therapy-oriented group intervention. American Journal of Psychiatry 158, 638–640. 11282702 10.1176/appi.ajp.158.4.638

[ref77] Zlotnick C , Miller IW , Pearlstein T , Howard M and Sweeney P (2006) A preventive intervention for pregnant women on public assistance at risk for postpartum depression. American Journal of Psychiatry, 163, 1443–1445. 16877662 10.1176/appi.ajp.163.8.1443 PMC4387544

